# Cyanide Toxicity to *Burkholderia cenocepacia* Is Modulated by Polymicrobial Communities and Environmental Factors

**DOI:** 10.3389/fmicb.2016.00725

**Published:** 2016-05-18

**Authors:** Steve P. Bernier, Matthew L. Workentine, Xiang Li, Nathan A. Magarvey, George A. O'Toole, Michael G. Surette

**Affiliations:** ^1^Department of Medicine, Faculty of Health Sciences, Farncombe Family Digestive Health Research Institute, McMaster UniversityHamilton, ON, Canada; ^2^Department of Biochemistry and Biomedical Sciences, Faculty of Health Sciences, McMaster UniversityHamilton, ON, Canada; ^3^Department of Microbiology and Immunology, Geisel School of Medicine at DartmouthHanover, NH, USA

**Keywords:** competition, cyanide, polymicrobial, community, metabolites, interactions

## Abstract

Microbes within polymicrobial communities can establish positive and negative interactions that have the potential to influence the overall behavior of the community. *Pseudomonas aeruginosa* and species of the *Burkholderia cepacia* complex (Bcc) can co-exist in the lower airways, however several studies have shown that *P. aeruginosa* can effectively kill the Bcc *in vitro*, for which hydrogen cyanide (HCN) was recently proposed to play a critical role. Here we show that modification of the environment (i.e., culture medium), long-term genetic adaptation of *P. aeruginosa* to the cystic fibrosis (CF) lung, or the addition of another bacterial species to the community can alter the sensitivity of *Burkholderia cenocepacia* to *P. aeruginosa* toxins. We specifically demonstrate that undefined rich media leads to higher susceptibility of *B. cenocepacia* to *P. aeruginosa* toxins like cyanide as compared to a synthetic medium (SCFM), that mimics the CF lung nutritional content. Overall, our study shows that the polymicrobial environment can have profound effects on negative interactions mediated by *P. aeruginosa* against *B. cenocepacia*. In fact, evolved *P. aeruginosa* or the presence of other species such as *Staphylococcus aureus* can directly abolish the direct competition mediated by cyanide and consequently maintaining a higher level of species diversity within the community.

## Introduction

Microbes rarely live alone in their natural environments but typically grow as part of multispecies communities (Curtis et al., 2002; Hall-Stoodley et al., 2004), an observation first made by Louis Pasteur (Pasteur and Joubert, 1877). This scenario is also true of infections, the majority of which are now recognized as polymicrobial (Peters et al., 2012). The long-term development of chronic polymicrobial infections in the lower airways is a common feature of obstructive lung diseases such as cystic fibrosis (CF), chronic pulmonary obstructive disease (COPD), and asthma (Huang and Lynch, 2011; Han et al., 2012; Dickson et al., 2014; Surette, 2014). Polymicrobial communities made of *Pseudomonas aeruginosa* and representative bacterial species from the non-pathogenic normal microbiota showed enhanced disease severity in different model systems compared to their monospecies counterparts (Duan et al., 2003; Sibley et al., 2008; Korgaonkar et al., 2013) demonstrating that interspecies interactions play a key role in disease progression. Co-infection by multiple primary pathogens is also common in the lower airways where bacterial pathogens like *P. aeruginosa, Staphylococcus aureus, Burkholderia cepacia* complex (Bcc), *Stenotrophomonas maltophilia, Streptococcus* milleri/anginosus group, and *Haemophilus influenzae* can co-exist (Harrison, 2007; Lipuma, 2010; Han et al., 2012; Surette, 2014).

Mixed *P. aeruginosa* and Bcc infections have been described in the lower airways of patients suffering from CF (Jacques et al., 1998; Lambiase et al., 2006; Schwab et al., 2014) and COPD (Han et al., 2012), wherein disease severity was reported to be worse than mono-infections in both humans (Jacques et al., 1998) and mice (Bragonzi et al., 2012) suggesting *in vivo* synergistic interactions or additive effects. Despite the fact that both pathogens can co-exist in the lower airways, several studies have reported that *P. aeruginosa* outcompetes and/or kills species of the Bcc both *in vitro* (Tomlin et al., 2001; Al-Bakri et al., 2004; Bakkal et al., 2010; Bragonzi et al., 2012; Costello et al., 2014; Rüger et al., 2014; Schwab et al., 2014; Smalley et al., 2015) and in a chronic lung infection mouse model (Bragonzi et al., 2012). Consequently, these observations strongly suggest that in mixed communities the fitness of *P. aeruginosa* is greater than *Burkholderia*, which may play a critical role in co-infections in terms of disease progression (Bragonzi et al., 2012). *In vitro* studies have suggested that pyocins, phenazines, rhamnolipids, hydrogen cyanide (HCN), the siderophore pyoverdine and other molecules yet to be identified are released by *P. aeruginosa* as a multifactorial strategy to outcompete or kill Bcc species (Tomlin et al., 2001; Bakkal et al., 2010; Costello et al., 2014; Smalley et al., 2015). However, co-infections for which Bcc species are numerically dominant over *P. aeruginosa* in the CF lungs (Schwab et al., 2014; Carmody et al., 2015) would argue against a model where Bcc species are always outcompeted by *Pseudomonas*. In fact, *in vitro* co-cultures with *Burkholderia multivorans* showed that anaerobic growth reduced the competitive advantage of *P. aeruginosa* and consequently increased *Burkholderia* survival compared to aerobic conditions (Schwab et al., 2014). The latter supported the hypothesis that environmental conditions might impact competitive interactions between *P. aeruginosa* and the Bcc by affecting *Pseudomonas* toxicity, *Burkholderia* tolerance, or both. Consequently, to better appreciate the *in vivo* dynamics of mixed *P. aeruginosa* and Bcc infections and their long-term consequences on disease progression, we must improve our *in vitro* molecular understanding of microbial interactions in the context of polymicrobial communities, including competition.

In the current study, we aim to expand our understanding of competitive interactions in mixed communities of *P. aeruginosa* and species of the Bcc. Herein, we first demonstrate that environmental factor(s) (i.e., growth medium) influence the competitive advantage of *P. aeruginosa* over *Burkholderia cenocepacia* (strain K56-2) in well-shaken aerobic co-cultures. More specifically, we show that the tolerance of *B. cenocepacia* K56-2 to cyanide and *P. aeruginosa*-derived metabolites is strongly affected by growth medium for which *B. cenocepacia* is more susceptible in rich media compared to chemically defined media. In addition to environmental factors, we show that the long-term adaptation of *P. aeruginosa* to the CF lung also has an impact on its toxicity where half of the *P. aeruginosa* isolates tested in co-culture pairs were dominated by *B. cenocepacia* within 24 h. However, although HCN-mediated toxicity is highly potent against the *Bcc*, it is not alone sufficient for *Burkholderia* killing in co-cultures as the less toxic quorum sensing (QS) *Pseudomonas* mutants Δ*lasR* and Δ*pqsA* and all tested *P. aeruginosa* CF adapted isolates were positive for the production of HCN but did not show complete killing, supporting a multifactorial killing model. Lastly, we show that co-cultures with cyanide-tolerant bacteria such as *S. aureus* and the less toxic QS *rhlR* mutant of *P. aeruginosa* allowed *B. cenocepacia* to survive and even grow at inhibitory concentrations of cyanide via the release of heat labile molecules(s) with *S. aureus* being the most protective.

## Materials and methods

### Bacterial strains, plasmids, chemicals and general growth conditions

Bacterial strains and plasmids used in this study are described in Table S1. Bacterial strains were routinely grown in Lysogeny broth (LB; Miller recipe) or on 1.5% LB-Miller agar (EMD Chemicals Inc., Gibbstown, NJ, USA) and incubated at 37°C. BHI (Becton, Dickinson and Company [BD], Sparks, MD, USA), SCFM (Palmer et al., 2007), and M9 minimal medium (BD) containing 0.5% glucose (M9_Glc_) or 0.5% casamino acids (M9_CAA_; [BD]) were also used where specified in the text. BHI and LB media were diluted with water to make them less rich in nutrients (1/4 or 1/10). Antibiotics were supplemented to the culture medium of *P. aeruginosa* strains when appropriate at the following concentrations: tetracycline (Tet), 150 μg ml^−1^; gentamicin (Gm), 50 μg ml^−1^; trimethoprim (Tp), 100 μg ml^−1^; ceftazidime (Caz), 32 μg ml^−1^; and ciprofloxacin (Cpx), 5 μg ml^−1^. Phenazine-1-carboxylic acid (PCA) was purchased from Princeton BioMolecular Research (Princeton, NJ, USA; Catalog no. PBMR030094) and dissolved in dimethyl sulfoxide (DMSO) at a final concentration of 2 mg ml^−1^. Pyocyanin was purchased from Sigma-Aldrich (St. Louis, MO, USA; Catalog no. P0046) and dissolved in DMSO at a final concentration of 10 mg ml^−1^. Potassium cyanide (KCN) was purchased from Sigma-Aldrich (Catalog no. 11813). Stock solutions of KCN (1 M in water) were diluted to 250 mM using 1 M MOPS buffered to pH 7.0 and filter sterilized using 0.2 μm filters (Millipore, Billerica, MA, USA). All other chemicals used in this study were obtained from Sigma-Aldrich.

### Monoculture and co-culture growth, extraction of spent medium, and bacterial survival quantification

Bacterial cells from overnight cultures (4 ml of LB medium in borosilicate glass test tubes) were spun down (2 min, 8000 rpm) and washed once in phosphate buffered saline (PBS) and used for inoculating liquid cultures in shaken flasks. *(i) Monoculture and co-culture (flasks)*. Flasks containing 25 ml of culture medium were inoculated with 80 μl of washed cells (1:300 dilution) and incubated for 24 h at 37°C with shaking (175 rpm). *(ii) Bacterial survival quantification*. Viable cell counts were used to determine bacterial growth or survival of both monoculture and co-culture growth. Bacterial cultures were serially diluted and plated on agar-containing medium with a detection limit of 200 colony forming units (CFUs) ml^−1^. For mixed cultures, selective agar media and colony morphotypes were used for accurate CFU counts for each bacterial species. To accurately quantify Bcc viable counts in co-cultures, LB agar supplemented with Gm (50 μg ml^−1^) and/or Columbia naladixic acid (CNA) agar plates were used. CFU counts for *P. aeruginosa* PA14 derivative strains were directly determined by colony morphotype on non-selective agar plates since their colonies are bigger and shiny compared to those from *B. cenocepacia* K56-2 that are smaller and rough (Bernier et al., 2008). To determine viable CFUs of *P. aeruginosa* clinical isolates in co-cultures, a combination of selective LB agar containing Caz (32 μg ml^−1^), Cpx (5 μg ml^−1^) or Tp (100 μg ml^−1^) in addition to Gm and CNA agar plates were used. *(iii) Extraction of bacterial spent media*. Bacterial cells from 25-ml cultures in flasks (24 h) were spun down and supernatant was filtered using 0.2 μm filters (Millipore) and used immediately or stored at 4°C. *(iv) Monoculture growth in 96-well plates*. Overnight cultures were diluted to an OD_600_ of 0.1 and 0.5 μl of the diluted culture was added to 100 μl of culture medium per well (1:200 dilution) in standard 96-well microtiter dish plate and incubated 37°C for 24 h with constant shaking in a plate reader (Synergy^TM^ H1, BioTek® Instruments, Inc., Winooski, VT) and optical density measurements (OD_600_) were taken every 30 min. To avoid evaporation, 65 μl of filtered mineral oil was added on top of the bacterial culture in each well.

### Determination of the minimal inhibitory concentration (MIC) and the fractional inhibitory concentration index (FICI)

MIC values of small molecules and spent media were determined by macrodilutions in LB as previously described (Hacek et al., 1999) with minor modifications. FICI values were determined as previously described (Odds, 2003; Pillai et al., 2005). Briefly, overnight cultures were diluted to an OD_600_ of 0.1 and added to 96-well plates at a final dilution of 1:200 (v/v). The final volume per well was 100 μl including the test compound and/or supernatant serially diluted. Inoculated plates were sealed with a sticky breathable membrane (Breathe-Easy, USA Scientific, Cat No. 9123-6100) and incubated for 24 h at 37°C with shaking (175 rpm).

### Genetic manipulations

Plasmids constructed in this study (Table S1) were made using homologous recombination in *Saccharomyces cerevisiae* as previously described (Shanks et al., 2006). Restriction enzymes and Phusion High-Fidelity DNA polymerase were obtained from New England BioLabs (Ipswich, MA, USA). All newly constructed plasmids were sequenced (MOBIX Lab, McMaster University, Hamilton, ON) using the universal primers M13 forward and reverse for verification (*i) Deletion of hcnABC in P. aeruginosa*. Briefly, two discontinuous fragments with overlapping sequences from primer design were PCR amplified with the primer pairs hcn1-5L hcn1-3 and hcn2-5 hcn2-3L (Table S1). The PCR fragments were subsequently cloned into plasmid pMQ30 (linearized with SmaI) via homologous recombination in *S. cerevisiae*. The resulting plasmid pMQ30*hcn*KO was transformed into *E. coli* SM10 and used to conjugate *P. aeruginosa* for ~6 h on LB agar and transconjugants were selected on *Pseudomonas* Isolation Agar (PIA; BD) containing Gm at 100 μg ml^−1^. Excision of the plasmid was performed onto LB agar (no NaCl) containing 5 % sucrose (w/v) at 30°C. Confirmation of the mutant genotype was performed by PCR using the primers hcnV5 and hcnV3 (Table S1) on Gm^*S*^ clones. *(ii) Construction of an HCN-expressing vector*. To build a plasmid expressing HCN, we amplified by PCR *hcnABC* from *P. aeruginosa* PA14 using primers hcnABC-5L and hcnABC-3L (Table S1). This fragment was cloned into SmaI-digested pMQ72 via homologous recombination in *S. cerevisiae*. This vector harbors the arabinose-inducible promoter system of *E. coli* (*P*_*BAD*_-*araC*), therefore addition of arabinose (0.1–0.4%) allows the induction of the *hcnABC* operon and production of HCN.

### Detection of volatile hydrogen cyanide and rhamnolipids

*(i) Hydrogen cyanide*. Detection of HCN was performed as previously described (Castric and Castric, 1983) with minor modifications. Briefly, overnight cultures were transferred into 384-well plates containing 75 μl of LB per well using pin replicator (VP408; vol. 0.2 μl—V&P Scientific, San Diego, CA, USA). The inoculated 384-well plates were then covered with dried Whatman chromatography paper previously soaked into the HCN detection reagent containing copper(II) ethyl acetoacetate (5 mg) and 4,4′-methylenebis-(*N,N*-dimethylaniline) (5 mg) in chloroform (1–2 ml). The HCN detection paper was subsequently sealed with a sticky breathable membrane (Breathe-Easy, USA Scientific) and incubated for 24 h at 37°C with shaking (175 rpm). For quantitative values of volatile HCN, the mean intensity of each colored spot on the membrane was measured with the ImageJ software and reported as relative units. *(ii) Rhamnolipids*. Detection of rhamnolipids was performed as previously described (Pinzon and Ju, 2009) with minor modifications. Briefly, 3 μl of an overnight culture was spotted onto agar plates containing methylene blue and cetyl trimethylammonium bromide (CTAB) and incubated for 24 h at 37°C and subsequently at 4°C for 24 h.

### Extraction and purification of rhamnolipids

Two liters of *P. aeruginosa* PA14 were grown in LB medium at 37°C with constant shaking (250 rpm) for 16–18 h. Cell-free supernatant was extracted by liquid-liquid extraction with equal volume of EtOAc. The organic extracts were concentrated on a rotary evaporator and analyzed by thin layer chromatography (TLC) on silica gel 60 plates (Merck). The column (2.5 cm × 35 cm) was packed with silica gel in chloroform and not allowed to dry. The sample was applied on the surface of the column and eluted with chloroform/methanol (v/v = 50:1, 20:1 and 10:1). Each fraction was evaporated on a rotary evaporator and analyzed by HPLC-MS/MS by using Bruker amaZon X ion trap mass spectrometer (operating in autoMS(2) positive ESI mode, scanning m/z 100–1800) coupled with a Dionex UltiMate 3000 HPLC, with an Ascentis Express C18 column (150 mm 4.6 mm, 2.7 m, Sigma-Aldrich) and A (acetonitrile with 0.1% formic acid) and B (water with 0.1% formic acid) as the mobile phases at 1 ml min^−1^. The solvent gradient was 0–45 min, 5% -100%A. Rhamnolipids containing fractions were further purified by Sephadex LH-20 and eluted with MeOH. Pure rhamnolipids were characterized by 1D and 2D NMR as well as MS/MS analysis.

### Statistical analysis

Two-tailed unpaired *t*-test was performed on log-transformed CFU values using Prism 5.0 for Mac OS X (GraphPad Software, Inc.). Spearmans' correlations coefficient (ρ) was performed on log-normalized data prior to performing the correlations.

## Results

### Hydrogen cyanide is necessary, but not sufficient for Bcc killing in co-cultures

**Figure 1:**
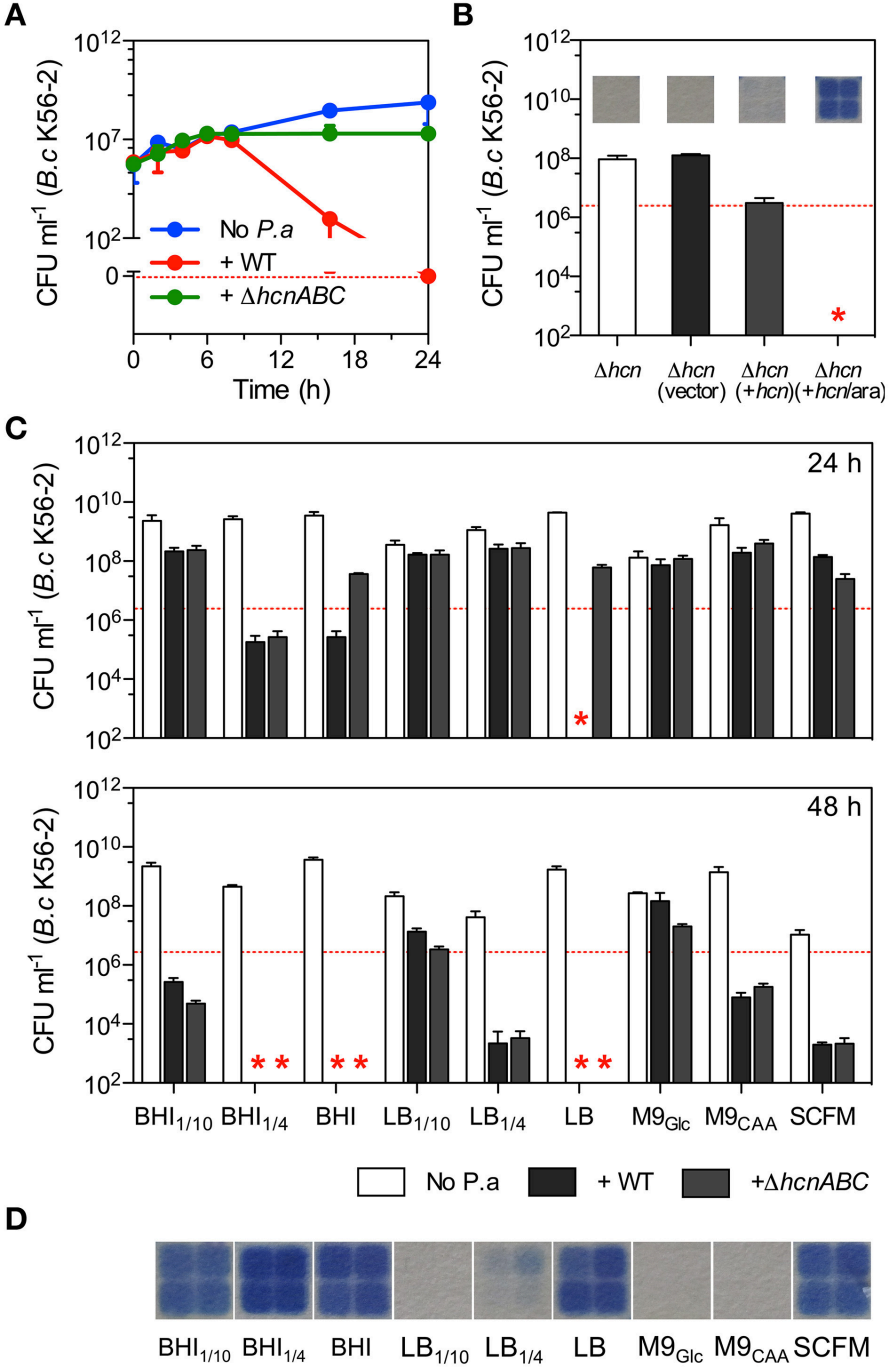
**Hydrogen cyanide-mediated killing is sensitive to growth conditions. (A)** Growth of *B. cenocepacia* K56-2 in monoculture (blue line) and in co-culture with WT (red line) *P. aeruginosa* (strain PA14) or its HCN-deficient mutant strain Δ*hcnABC* (green line) over 24 h in LB shaken cultures. **(B)** Complementation *in trans* of Δ*hcnABC* (Δ*hcn*) with pMQ72*hcnABC* (+*hcn*) when induced with 0.1% arabinose (+*hcn*/ara) fully restored *B. cenocepacia* K56-2 killing in LB co-cultures. **(C)** Release of volatile HCN by WT *P. aeruginosa* (strain PA14) in different culture media and the survival of *B. cenocepacia* (strain K56-2) in co-cultures after 24 (top) and 48 h (bottom) with WT *P. aeruginosa* (black bars) or Δ*hcnABC* (gray bars). **(D)** Detection of volatile HCN from *P. aeruginosa* PA14 cultures in different growth media. Data reported represent the mean ± SD of a minimum of three replicates. Dotted red line in **(A)** represents the 0 value while it represents CFUs of *B. cenocepacia* K56-2 at time 0 (~2 × 10^6^ CFU ml^−1^) in **(B,C)**. Red stars in **(B,C)** represent the absence of *B. cenocepacia* K56-2 CFU recovered from the co-cultures or below the detection limits.

Smalley et al. (2015) recently showed that *P. aeruginosa*-derived HCN was an important toxin for killing *B. multivorans* in co-cultures. Using LB medium, we genetically re-confirmed that deletion of the HCN operon (Δ*hcnABC*) was sufficient to reduce the advantage of *P. aeruginosa* (strain PA14) in large shaken LB co-cultures (i.e., flasks) within 24 h with different *B. multivorans* strains as well as with other Bcc species namely *B. cenocepacia* and *Burkholderia dolosa* (Figure 1A and Figure S1). Genetic complementation with a functional *hcnABC* under an arabinose inducible promoter fully restored the ability of Δ*hcnABC* to produce volatile HCN and to kill *B. cenocepacia* (strain K56-2) in LB co-cultures (Figure 1B) confirming the role of the HCN operon in Bcc killing. Deletion of the *hcnABC* operon in strain PAO1 was also impaired for killing in LB co-cultures (Figure S2). Our results along with those from Smalley et al. (2015) suggest that *Pseudomonas* biogenic HCN is important for killing not only *B. multivorans*, but also other Bcc species in LB co-cultures demonstrating the broad sensitivity of the Bcc to cyanide.

To next determine whether *P. aeruginosa* could also kill *Burkholderia* via the release of HCN in non-LB media, we co-cultured *B. cenocepacia* K56-2 and *P. aeruginosa* PA14 in undefined-rich BHI and the chemically defined media M9_Glc_, M9_CAA_, and SCFM (Palmer et al., 2007). Although SCFM mimics the nutritional content of CF sputum and supports the growth of *P. aeruginosa* (Palmer et al., 2007) and *B. cenocepacia* (McKeon et al., 2011), *S. aureus* poorly grows in SCFM (Figure S3) demonstrating that this medium lacks components required for the growth of some common pathogens found in the CF airways. In addition to those media, we also co-cultured *B. cenocepacia* and *P. aeruginosa* in diluted LB and BHI (LB_1∕10_, LB_1∕4_, BHI_1∕10_, BHI_1∕4_). Unlike undiluted LB, no other culture media led to complete eradication of *B. cenocepacia* within 24 h in mixed cultures (Figure 1C). However, complete killing occurred within 48 h for co-cultures in BHI and BHI_1∕4_ while a constant increase in *Burkholderia* killing was observed for all other tested media over a period of 48 h with the exception of M9_*Glc*_ (Figure 1C). However, although the release of biogenic volatile HCN by WT *P. aeruginosa* PA14 was detected in the majority of the tested culture media with the exception of LB_1∕10_, M9_*Glc*_, and M9_*CAA*_ (Figure 1D) co-cultures with Δ*hcnABC* led to increased viability of *B. cenocepacia* K56-2 compared to the WT parent PA14 only in LB and BHI while minimal differences occurred with the other media over a period of 24 h (Figure 1C). Interestingly, survival of *B. cenocepacia* with Δ*hcnABC* in both LB and BHI was completely lost within 48 h (Figure 1C) demonstrating that other toxic molecules reached inhibitory concentrations after 24 h. Accumulation of inhibitory concentrations of non-HCN toxic metabolites over time was not due to continued growth since the viable population of *P. aeruginosa* was similar between 24 and 48 h (Figure S4).

Altogether, our results demonstrate that HCN production is necessary, but not sufficient for complete *B. cenocepacia* killing in large shaken planktonic cultures. Although co-culture in undiluted LB medium was the only condition in which *B. cenocepacia* K56-2 was eradicated within 24 h (Figure 1C), our results suggest that competition between *P. aeruginosa* and Bcc species in liquid co-cultures is both medium- and growth phase-dependent (Figure 1 and Figure S5), for which conditions reached in rich media (i.e., LB and BHI) seem to be favorable for effective *Burkholderia* killing. In addition, accumulation of *P. aeruginosa* non-HCN toxic metabolites over time is sufficient for complete *Burkholderia* killing in some, but not all growth media.

### The susceptibility of *B. cenocepacia* K56-2 to cyanide and other *P. aeruginosa*-derived toxins is greater in undefined rich culture media

The lack of efficient HCN-mediated killing by *P. aeruginosa* PA14 in chemically defined or diluted rich media within 24 h (Figure 1C) compared to rich LB medium could be the result of lower toxin concentrations (i.e., sub-inhibitory) and/or increased *Burkholderia* tolerance to cyanide and other toxic molecules in these particular conditions. To address the latter, we first determined the minimal inhibitory concentration (MIC) of potassium cyanide (KCN), as a source of cyanide, in culture media for which volatile HCN was produced and detected.

Using 96-well microtiter plates, we first showed that the susceptibility of *B. cenocepacia* K56-2 to KCN was greatly affected by culture medium with MICs spanning from 0.4 to > 25 mM. Assays done in undiluted rich media (i.e., BHI and LB) gave the lowest MICs and those done in synthetic media (SCFM) gave the highest MICs (Table 1). The high cyanide susceptibility in LB medium was further demonstrated within the Bcc by testing four *B. multivorans* strains (C1576, C5393, CF-A1-1, and ATCC 17616) and one *B. dolosa* strain (PC543) with MIC values for KCN between 1.6 and 3.2 mM in comparison to 12.5 and >25 mM for *P. aeruginosa* PA14 and *S. aureus* (strain RN6390), respectively (Table 2). Growth profiles of *B. cenocepacia* K56-2 were different in these culture media, which may explain some of the differences in susceptibility to cyanide. In fact, the lag phase was longer in chemically defined media compared to rich media like LB and BHI (Figure S6).

**Table 1:** **Susceptibility of *B. cenocepacia* K56-2 to *P. aeruginosa*-derived small molecules**.

**Medium**	**Spent medium (%)^a^**	**KCN (mM)^b^**	**PYO (μg ml^−1^)^b^**	**PCA (μg ml^−1^)^b^**	**RLs (μg ml^−1^)^b^**
LB	50	0.8	>300	>60	≥600
LB_1∕4_	>50	1.6	>300	>60	N.D.
LB_1∕10_	50	3.2	>300	>60	N.D.
BHI	>50	0.4	>300	7.5	N.D.
BHI_1∕4_	>50	1.6	>300	>60	N.D.
BHI_1∕10_	12.5	1.6	>300	>60	N.D.
M9_Glc_	12.5	N.D.	N.D.	N.D.	N.D.
M9_CAA_	12.5	N.D.	N.D.	N.D.	N.D.
SCFM	25	>25	>300	>60	N.D.

*MIC values after 24 h of growth in 96-well format using B. cenocepacia K56-2 are reported*.

a *Spent medium was extracted from 24-h planktonic cultures of WT P. aeruginosa PA14 grown as described in the Materials and Methods section. The antimicrobial activity of each spent medium was determined in the same growth medium for which supernatants were extracted*.

b *The MIC of purified compounds were determined in LB medium*.

*N.D., Not determined; KCN, potassium cyanide; PYO, pyocyanin; PCA, phenazine-1-carboxylic acid; RLs, di-rhamnolipids*.

**Table 2:** **Cyanide susceptibility of CF associated bacterial pathogens**.

**Species**	**Strain**	**KCN (mM)**
*B. multivorans*	C1576	1.6
	C5393	3.2
	CF-A1-1	1.6
	ATCC 17616	1.6
*B. cenocepacia*	K56-2	0.4–0.8
*B. dolosa*	PC543	3.2
*P. aeruginosa*	PA14	12.5
*S. maltophilia*	K279a	0.8
*S. aureus*	RN6390	>25

*MIC values after 24 h of growth are reported. Bacteria were grown in LB medium using 96-well microtiter plates and incubated aerobically at 37°C with constant shaking (175 rpm)*.

Although the susceptibility of *B. cenocepacia* K56-2 to cyanide was highly dependent on culture media (Figure 1C and Table 1), the bioactivity of other *P. aeruginosa* toxins (Figure S1 and Tomlin et al., 2001; Bakkal et al., 2010; Costello et al., 2014; Smalley et al., 2015) could also be affected which may further explain the lack of killing in cyanide-positive conditions (Figures 1C,D). To address this possibility, we extracted supernatants from 24-h monocultures of *P. aeruginosa* PA14 grown in all nine culture media (i.e., LB, LB_1∕4_, LB_1∕10_, BHI, BHI_1∕4_, BHI_1∕10_, M9_Glc_, M9_CAA_, and SCFM) previously tested in co-cultures (Figure 1C) to determine their respective antibiotic-like activity. All tested *P. aeruginosa* spent media had different levels of toxicity on the growth of *B. cenocepacia* K56-2 with MIC values spanning from 12.5 to greater than 50% where supernatants from the chemically defined M9 and SCFM being the most toxic (Table 1). In addition, the MIC of spent medium from Δ*hcnABC* LB cultures was similar (~25–50%) to its WT parent demonstrating that non-HCN toxins are active against *Burkholderia* in co-cultures (Figure 1C and Figure S1). Furthermore, since HCN is highly volatile, its concentration in spent medium after extraction is likely sub-inhibitory. These results suggested that the toxicity of *Pseudomonas* was maintained in most culture media; including those from chemically defined M9 or SCFM that were not highly toxic in co-cultures within 24 h (Figure 1C).

The increased susceptibility of *B. cenocepacia* to *P. aeruginosa* and cyanide in rich medium may suggest that the susceptibility of *Burkholderia* may vary in function of the growth medium. To demonstrate this possibility, we determined the MIC of *Pseudomonas* spent medium from SCFM cultures (low MIC; Table 1) in LB medium as well as *Pseudomonas* spent medium from LB cultures in SCFM medium. Interestingly, the MIC of SCFM spent medium required to inhibit the growth of *B. cenocepacia* K56-2 went from 25% in SCFM to less than 6.25% in LB medium while the MIC of LB spent medium stayed at 50% in both culture media. These results demonstrated that higher amounts of *Pseudomonas* toxins were present in SCFM compared to LB cultures since the MIC of those in LB medium was much lower than in SCFM confirming that *B. cenocepacia* is more susceptible to *P. aeruginosa* toxins in rich medium like LB than SCFM. Other toxins like phenazines and rhamnolipids were shown using mutant strains to have antibiotic-like activity in LB co-cultures against *B. multivorans* (Smalley et al., 2015), which we re-confirmed in other Bcc species (Figure S1). However, purified pyocyanin (PYO) and phenazine-1-carboxylic acid (PCA), two phenazines produced by *P. aeruginosa* (Price-Whelan et al., 2006), did not exhibit much toxicity in all tested growth conditions with the exception of PCA in BHI (Table 1). In addition, purified di-rhamnolipids (RLs) did not have any antimicrobial activity against *B. cenocepacia* K56-2 in LB medium (Table 1). These results demonstrated that the toxicity of other potential small molecules could also be impacted by growth medium.

Overall, our results demonstrate that *Pseudomonas* toxic metabolites are present at inhibitory concentrations when grown in chemically defined media like SCFM, but the susceptibility of *B. cenocepacia* K56-2 to these chemicals, including cyanide, is greatly influenced by environmental factors (i.e., culture medium) for which *B. cenocepacia* is more sensitive in rich medium. Altogether, it demonstrates that the growing environment plays a critical role in the ability of *B. cenocepacia* to tolerate the toxicity of *P. aeruginosa* mediated by the release of small molecules such as cyanide and phenazines.

### The relationship between *P. aeruginosa* quorum sensing, hydrogen cyanide production and *burkholderia* killing

Loss-of-function mutations in genes involved in quorum sensing (QS) regulation are often isolated in host-adapted *P. aeruginosa* CF isolates (Smith et al., 2006; Hoffman et al., 2009; Cullen and McClean, 2015). *P. aeruginosa* uses three QS systems (Las, Rhl, and PQS) as global regulators to coordinate the regulation of target gene networks, including the *hcnABC* operon (Jimenez et al., 2012; Tashiro et al., 2013) and mutants in the Las or the Rhl QS system were shown to be less toxic for *B. multivorans* in LB co-cultures (Smalley et al., 2015) while PQS negative supernatants had no growth inhibition on *B. cenocepacia* (Costello et al., 2014). Here, we wanted to determine the contribution of each of the three QS systems with respect to *Burkholderia* killing and HCN production.

**Figure 2:**
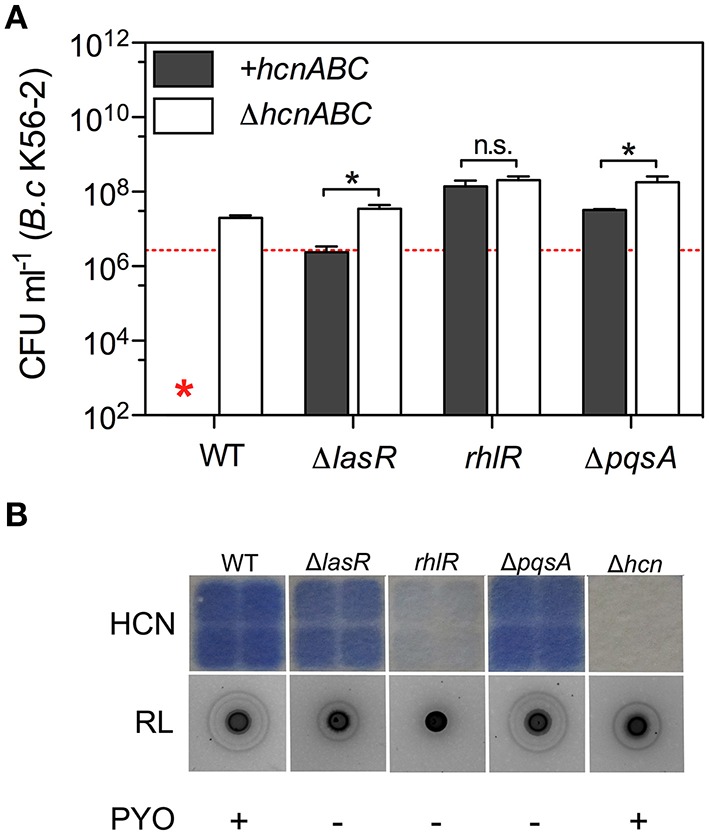
**Relationships between quorum sensing, HCN production, and *Burkholderia* killing. (A)** Viability of *B. cenocepacia* K56-2 after 24 h in mixed cultures WT *P. aeruginosa* PA14 and its QS-deficient mutant strains Δ*lasR, rhlR*, and Δ*pqsA* positive (gray bars; +*hcnABC*) or negative (white bars; Δ*hcnABC)* for the *hcnABC* operon. **(B)** Phenotypic profiling of *P. aeruginosa* PA14-derived strains tested in **(A)** for their *in vitro* ability to produce volatile HCN, rhamnolipids (RL), and pyocyanin (PYO). Red star represents the absence of *B. cenocepacia* K56-2 CFU recovered from the co-cultures or below the detection limits. Data reported represent the mean ± SD of a minimum of three replicates. Dotted red line in **(A)** represents *B. cenocepacia* K56-2 CFUs at time 0 (~2 × 10^6^ CFU ml^−1^). Asterisks indicate values significantly different by two-tailed unpaired *t*-test: ^*^P ≤ 0.001 and n.s. (not significant).

Where optimal killing occurs, in LB co-cultures, all tested Bcc strains representing *B. multivorans, B. cenocepacia*, and *B. dolosa* showed increased survival within 24 h in the presence of any of the three QS-deficient mutants (Δ*lasR, rhlR*, and Δ*pqsA*) compared to their wild-type (WT) parent (Figure 2A and Figure S7). Introduction of the Δ*hcnABC* mutation into Δ*lasR* and Δ*pqsA* mutant strains significantly increased the viability of *B. cenocepacia* K56-2 while no difference was observed when the same mutation was added into *rhlR* mutant strain (Figure 2A). In agreement with these results, both Δ*lasR* and Δ*pqsA* still produced HCN unlike *rhlR* for which the levels of detected volatile HCN were severely reduced compared to WT (Figure 2B). To next determine whether survival of *B. cenocepacia* K56-2 in co-cultures with Δ*pqsA* was mediated via PQS or 2-heptyl-4-quinolone (HHQ), we tested a Δ*pqsH* mutant strain. HHQ, the precursor of PQS, is produced by the *pqsABCDE* operon, while PqsH further converts HHQ into PQS (Déziel et al., 2004; Tashiro et al., 2013). Unlike Δ*pqsA* (i.e., HHQ negative, PQS negative), Δ*pqsH* (i.e., HHQ positive, PQS negative) was still fully toxic in co-cultures while Δ*pqsR* (i.e., HHQ negative, PQS negative), the positive regulator of the operon was not (Figure S8). HHQ and PQS can bind the PqsR regulator to coordinate the expression of target genes (Jimenez et al., 2012), consequently our genetic results can only confirm that mechanism(s) of *Burkholderia* killing regulated by the PQS system are PqsR-dependent since we did we did not test a mutant strain that was PQS positive and HHQ negative to separate the two specific pathways.

Interestingly, disruption of *rhlR* allowed all tested Bcc strains to reach cell densities close to those normally achieved during monoculture growth, which was not always the case for co-cultures with Δ*lasR* or Δ*pqsA* mutant strains (Figure 2A and Figure S7). In all cases, the increased survival of *Burkholderia* was not due to the reduced ability of the QS-deficient mutants to compete in mixed cultures, but rather their reduced toxicity toward Bcc species, since viable counts of the *P. aeruginosa* strains were equivalent to those of the WT parent and comparable to monoculture growth (Figure S9). These results demonstrate that while *P. aeruginosa*-derived HCN is a major toxin for the Bcc, additional factors are required for effective killing that are LasR-, RhlR-, and PqsR-regulated such as phenazines, rhamnolipids (Figure 2B and Figure S1; Smalley et al., 2015), pyoverdine via iron chelation (Costello et al., 2014; Tyrrell et al., 2015), and other molecules yet to be identified.

### The *burkholderia* killing trait is diverse among host-adapted *P. aeruginosa* CF isolates

**Figure 3:**
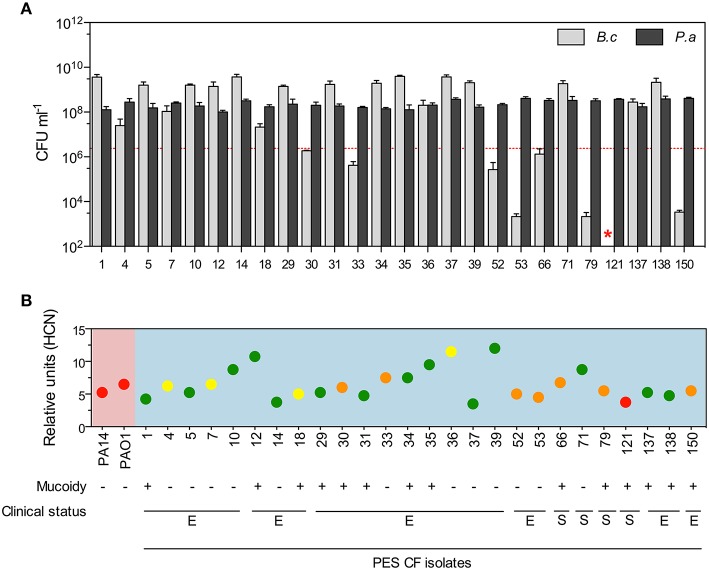
***Burkholderia* killing diversity among *P. aeruginosa* CF isolates. (A)** *B. cenocepacia* K56-2 was co-cultured in LB medium in pairs with 26 *P. aeruginosa* CF isolates collected from a single patient during different exacerbation (E) and clinically stable (S) periods where survival/growth of each species was monitored after 24 h. **(B)** Relative levels of volatile HCN produced by each PES isolates (blue background) compared to the laboratory strain PA14 and PAO1 (pink background) within 24 h in LB cultures and their ability to kill or not kill *B. cenocepacia* K56-2 in co-cultures are represented by the dots in green, yellow, orange, and red. The meaning of the colors are as follow: total eradication of *Burkholderia* (red), growth/survival of *Burkholderia* above (yellow) or below (orange) the initial inoculum (dotted red line in **A**), and higher numbers of *Burkholderia* compared to *P. aeruginosa* (green). Red star represents the absence of *B. cenocepacia* K56-2 CFUs recovered from the co-cultures or below the detection limits. Data reported in **(A)** represent the mean ± SD of a minimum of three replicates and the dotted red line represents *B. cenocepacia* K56-2 CFUs at time 0. Values for volatile HCN quantification (relative units) in **(B)** represent the mean ± SD of four replicates.

Pathogenic bacteria adapt and diversify phenotypically in polymicrobial infections of the lower airways (Cullen and McClean, 2015). *P. aeruginosa* has been shown to acquire numerous adaptive mutations in the airways (Smith et al., 2006) and to establish phenotypically diverse populations (Mowat et al., 2011; Workentine et al., 2013; Jorth et al., 2015). To assess whether the long-term adaptation to the CF lung may impact the toxicity of *P. aeruginosa*, we evaluated a collection of *Pseudomonas* CF isolates belonging to the Prairie epidemic strain (PES) (Parkins et al., 2014). We tested 26 representative clinical isolates that were obtained from a single patient over a period of 10 months during both exacerbation and clinically stable periods (Figure 3B) and for which phenotypic diversity (i.e., colony morphotypes, growth, motility, QS signals, siderophores, proteolytic activity, and antibiotic resistance) was previously shown (Workentine et al., 2013).

Using LB medium as optimal *Burkholderia* killing growth medium (Figure 1C), we co-cultured 26 *P. aeruginosa* CF isolates in pairs with *B. cenocepacia* K56-2 and found that only one isolate (#121) was able to eradicate *B. cenocepacia* from the mixed culture while others had intermediate to no toxicity (Figure 3A). Interestingly, half of the co-culture pairs were dominated by *B. cenocepacia* rather than *P. aeruginosa* after 24 h (Figures 3A,B; green circles) suggesting either neutral or positive interactions with these strains. All tested 26 *P. aeruginosa* CF isolates were capable of producing volatile HCN *in vitro* at similar or higher levels than those from the laboratory strains PA14 and PAO1 (Figure 3B). Statistical correlation analysis comparing *B. cenocepacia* K56-2 CFUs after 24 h in each co-culture pair was directly compared to previously evaluated phenotypes of the respective 26 *P. aeruginosa* PES isolates (Workentine et al., 2013) to find possible predictors of *Burkholderia* survival or killing. Spearman rank correlation did not show any statistical correlation of *B. cenocepacia* CFUs with HCN, QS signals (C4- and C12-HSL), or growth in LB (Figure S10).

Using a small subset of evolved *P. aeruginosa* CF isolates from a single patient, we demonstrated using simple pairwise co-cultures the full spectrum of competitive interactions that could possibly occur in complex *in vivo* communities where both species can dominate each other or establish population equilibrium. Further, HCN production alone did not explain *Burkholderia* killing in co-cultures with clinical isolates, however additional factors may be required for effective killing reinforcing the concept of multifactorial strategy.

### The composition of bacterial communities influences the tolerance of *B. cenocepacia* to cyanide

Chronic infections affecting the lower airways are polymicrobial (Huang and Lynch, 2011; Han et al., 2012; Dickson et al., 2014; Surette, 2014), consequently we next evaluated whether the presence of other CF pathogens namely *S. aureus* and *S. maltophilia* would impact the tolerance of *B. cenocepacia* K56-2 to cyanide in LB co-cultures. The tolerance of *S. maltophilia* (strain K279a) to cyanide (i.e., KCN) in LB was comparable to Bcc species while *S. aureus* (strain RN6390) was fully resistant (Table 2). The cyanide resistance profiles of these two pathogens span the concentration spectrum making these attractive candidates to use to determine their impact on *Burkholderia* cyanide tolerance in mixed communities.

**Figure 4:**
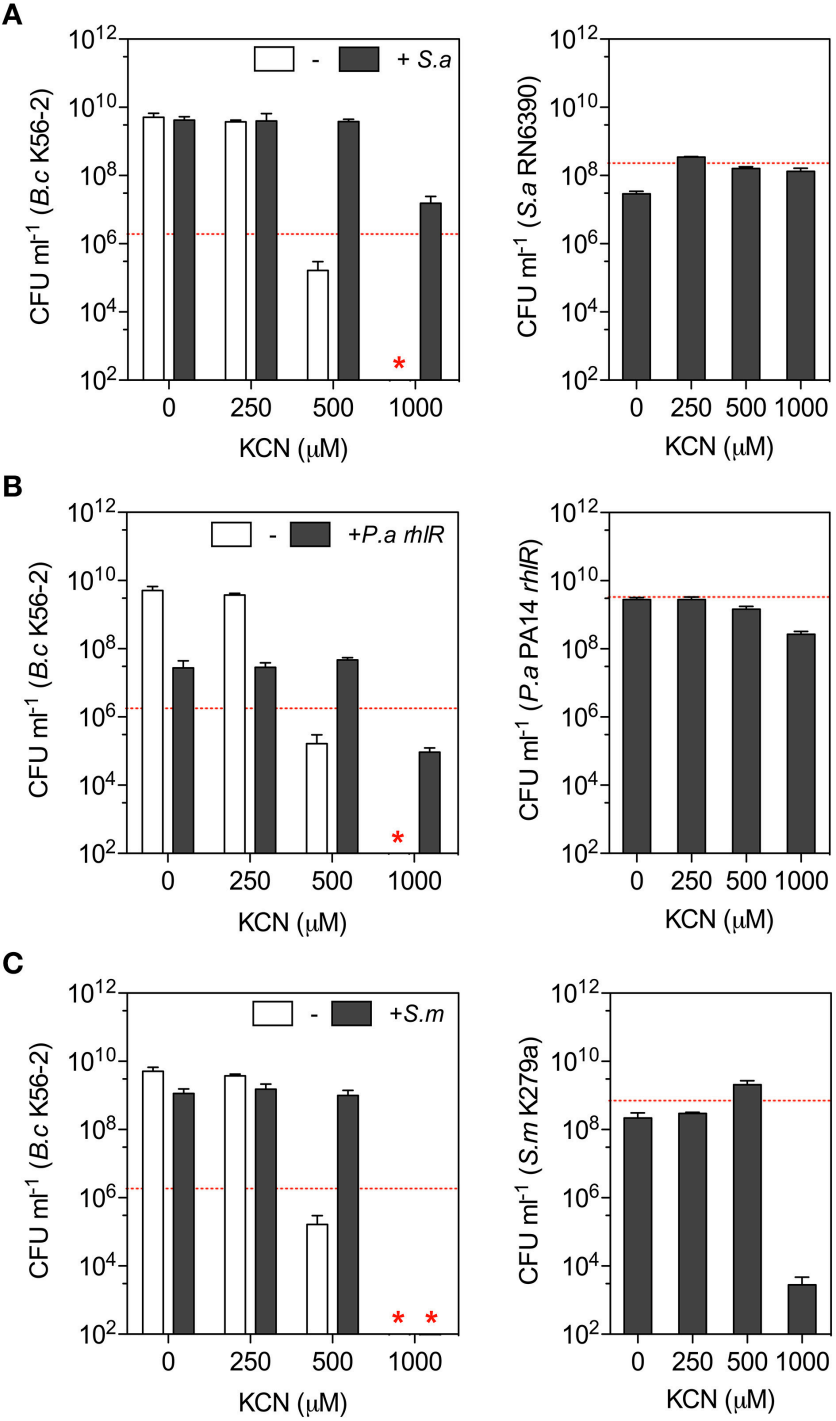
**The impact of community composition on *B. cenocepacia* cyanide tolerance**. *B. cenocepacia* K56-2 was co-cultured in LB medium with **(A)** *S. aureus* RN6390, **(B)** *P. aeruginosa rhlR*, and **(C)** *S. maltophilia* K279a for 24 h with increasing concentrations of KCN (0–1000 μM). Left panels: Viability of *B. cenocepacia* K56-2 after 24 h in the presence (gray bars) or the absence (white bars) of second bacterial species. Red star represents the absence of *B. cenocepacia* K56-2 CFU recovered from the co-cultures or below the detection limits and the dotted red lines represent *B. cenocepacia* K56-2 CFUs at time 0 (~2 × 10^6^ CFU ml^−1^). Right panels: Viability/growth of **(A)** *S. aureus* RN6390, **(B)** *P. aeruginosa rhlR*, and **(C)** *S. maltophilia* K279a for 24 h in co-cultures with *B. cenocepacia* K56-2 and KCN. The dotted red lines represent the biomass after 24 h of these bacteria in absence of both *B. cenocepacia* and KCN. Data reported represent the mean ± SD of a minimum of three replicates.

We monitored the viability of *B. cenocepacia* K56-2 in the presence of increasing concentrations of KCN (0–1000 μM) in monocultures and co-cultures with *S. aureus* RN6390, *S. maltophilia* K279a, and the less toxic *rhlR* mutant of *P. aeruginosa* strain PA14 after 24 h in LB medium. In monocultures, the growth/viability of *B. cenocepacia* K56-2 was severely impaired with 500 μM KCN while it was fully eradicated with 1000 μM (Figure 4). The addition of *S. maltophilia* increased the growth of *B. cenocepacia* K56-2 with 500 μM KCN (Figure 4C) while the addition of *S. aureus* or *rhlR* allowed *Burkholderia* to survive and grow with KCN concentrations up to 1000 μM (Figures 4A,B). At the species level, the cyanide tolerance observed in co-cultures reflected what was previously determined in monocultures with *S. aureus* being the most tolerant followed by *P. aeruginosa, S. maltophilia*, and *B. cenocepacia* (Table 2 and Figure 4). In agreement with the latter, the viability of *B. cenocepacia* in co-cultures with inhibitory concentrations of KCN followed a similar trend with *S. aureus* being the most protective against cyanide toxicity (Figure 4).

**Figure 5:**
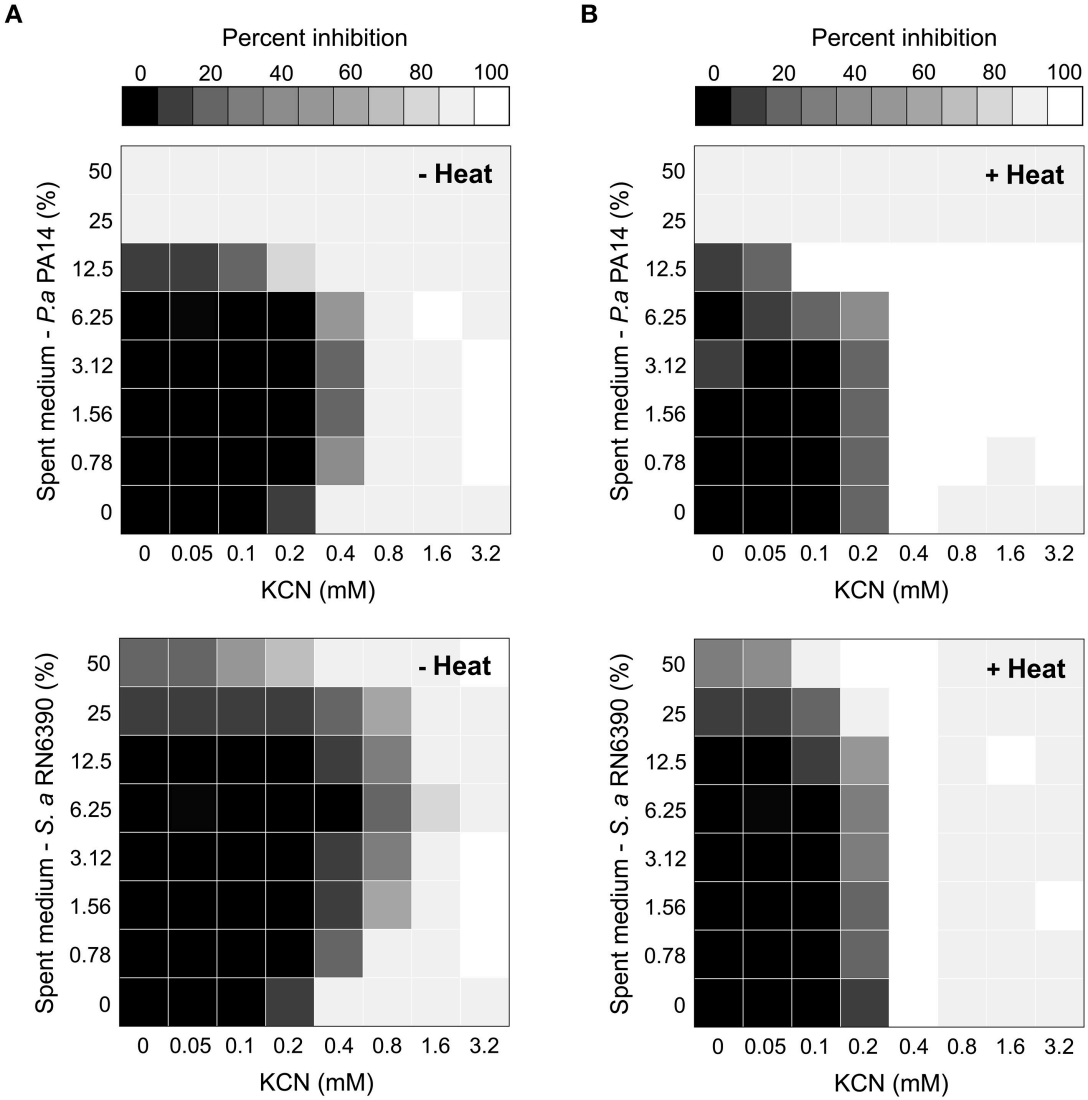
**The antagonistic effect of *P. aeruginosa* and *S. aureus* supernatants on the antimicrobial activity of potassium cyanide against *B. cenocepacia***. Heat plots showing the antagonistic growth effect of *P. aeruginosa* PA14 (top) and *S. aureus* RN6390 (bottom) spent media on the toxicity of KCN on the growth of *B. cenocepacia* K56-2 before **(A)** and after **(B)** heat treatments. Antagonism mediated by heat labile molecule(s) was evident as the MIC for KCN increased with untreated spent medium while being unaffected after heat treatments.

These results suggested that molecule(s) released in the extracellular milieu by other bacterial species could allow *Burkholderia* to tolerate higher concentrations of KCN. To test this hypothesis and the potential antagonistic effects between bacterial spent medium and KCN, we used the fractional inhibitory concentration index (FICI) in checkerboard liquid cultures as a measure of the interaction between two antimicrobial agents (Odds, 2003; Pillai et al., 2005). FICI of ≤ 0.5, > 4.0, and > 0.5–4.0 indicate synergy, antagonism, and no interaction, respectively (Odds, 2003). Addition of spent medium from *P. aeruginosa* PA14 or *S. aureus* RN6390 increased the tolerance of *B. cenocepacia* K56-2 to cyanide with FICI of 4.0625 and > 8.125, respectively (Figure 5A), confirming the antagonistic interactions between supernatants from these two bacterial species and KCN. Spent medium from either *S. maltophilia* K279a or *B. cenocepacia* K56-2 in combination with KCN resulted in an FICI of 2.0625 and 0.75, respectively representing no detectable synergistic or antagonistic interaction. These results corroborated the co-culture observations (Figure 4) for which both *P. aeruginosa* PA14 and *S. aureus* RN6390 increased the cyanide tolerance of *B. cenocepacia* K56-2 in mixed communities with *S. aureus* being the most protective in both cases (Figures 4,5A). Heat treatment of *P. aeruginosa* PA14 and *S. aureus* RN6390 supernatants abolished the antagonistic interactions with FICI of 0.75 and 1.0, respectively (Figure 5), suggesting that heat labile extracellular molecule(s) from these bacterial species could be protective against cyanide. Altogether these results demonstrate that the overall composition of the microbial community can have profound effects on competitive mechanisms mediated by the exchange of toxic metabolites like cyanide.

## Discussion

Microbial interactions (e.g., positive and competitive) in polymicrobial infections can have profound effects on disease progression (Peters et al., 2012; Murray et al., 2014; Short et al., 2014) and antibiotic resistance (Bernier and Surette, 2013). Although, the concept of cooperation and polymicrobial synergy is well documented (Peters et al., 2012; Murray et al., 2014; Short et al., 2014) not much is known about the role of competition in mixed bacterial infections. Co-infections or succession of pathogens in the CF airways could involve interspecies competition and consequently change the composition of the community with the potential to affect the progression of the infection. As example, *P. aeruginosa* uses a multifactorial strategy to outcompete *S. aureus* in co-cultures (Filkins et al., 2015), which could explain, in certain cases, the replacement of *S. aureus* in CF airways at late adolescence and adulthood (Harrison, 2007).

In the current study, we focused on expanding our *in vitro* understanding of competitive interactions mainly mediated by cyanide that was recently proposed to be an important strategy used by *P. aeruginosa* to kill *B. multivorans* in LB co-cultures (Smalley et al., 2015). HCN is a poison produced by *P. aeruginosa* in the CF lung that can affect host immune functions (Anderson et al., 2010; Lenney and Gilchrist, 2011; Nair et al., 2014), with also the potential to outcompete other bacterial species in polymicrobial infections and consequently reduce species diversity of these complex communities. However, we showed that the competitive efficacy of *P. aeruginosa* toward the Bcc via the release of toxins, including cyanide, depends on several factors such as the growth medium, the *in vivo* adaptive evolution of *P. aeruginosa*, and the presence of other bacteria in the mixed community.

Deletion of the *hcnABC* operon increased the viability of all tested Bcc species in LB co-cultures with *P. aeruginosa* within 24 h (Figure 1 and Figure S1), however loss of HCN failed to maintain the viability of *B. cenocepacia* over 48 h in rich LB and BHI media (Figure 1C) suggesting that accumulation of non-HCN toxins reached inhibitory concentrations. These results are in agreement with the fact that Bcc killing is QS-mediated (Figure 2 and Figure S7; Costello et al., 2014; Smalley et al., 2015), which also involve the QS-regulated phenazines and rhamnolipids (Figure S1). Interestingly, *B. cenocepacia* was more susceptible to cyanide and other toxins (e.g., spent medium) in rich medium than in SCFM (Table 1). Although differences in nutrients between culture media may affect metabolism and consequently tolerance to *P. aeruginosa* toxins, we showed that the growth profile of *B. cenocepacia* was different with a longer lag phase in SCFM compared to LB and BHI media (Figure S6). An extended lag phase could impact the tolerance of *Burkholderia* to *P. aeruginosa* toxins, similarly to the increased tolerance of evolved *E. coli* populations due to longer lag phase (Fridman et al., 2014). Whether SCFM reflects the nutritional content of the CF lung is an important question, but knowing that growth of *S. aureus* is not supported in SCFM (Figure S3) suggests that key components are missing to support the growth of CF pathogens other than *P. aeruginosa* and the Bcc.

Biogenic HCN has pleiotropic biological roles since it can act as a classical virulence factor (Anderson et al., 2010; Zdor, 2015), a biocontrol agent of plant fungal diseases (Blumer and Haas, 2000; Zdor, 2015), a policing agent controlling the emergence of social cheaters (Wang et al., 2015), and results from our study suggest that it could be used as a strategy to outcompete other bacterial species in polymicrobial infections and consequently reduce species diversity of these complex communities. How the Bcc is killed by cyanide remains an unanswered question, but cyanide generally poisons susceptible eukaryotic and prokaryotic cells by interfering with the aerobic respiratory chain by inhibiting cytochrome c oxidase (Lenney and Gilchrist, 2011; Wybouw et al., 2014). To tolerate higher concentrations of cyanide, *P. aeruginosa* possesses cyanide-resistant oxidases and the rhodanese RhdA that converts cyanide into the less toxic thiocyanate (Cunningham and Williams, 1995; Cooper et al., 2003; Cipollone et al., 2007a,b). Results from our study and Smalley et al. (2015) conclusively demonstrate the high sensitivity of the Bcc to cyanide, but this is rather surprising since it was previously suggested that the Bcc produced HCN (Ryall et al., 2008; Neerincx et al., 2015). However, another study failed to detect volatile HCN from Bcc grown as biofilms or in liquid or from breath samples of *Burkholderia*-infected CF patients (Gilchrist et al., 2013). Although these studies are controversial, we did not detect any volatile HCN released from 41 strains representing nine Bcc species within 24 h in LB cultures (data not shown). Although 3 potential *hcnABC* homologs were found in *B. cenocepacia* (Ryall et al., 2008) and the discrepancy between different studies, further work is required to better understand the possible relationship between cyanide production and sensitivity in the Bcc.

As the competitive interactions described here occur mainly through metabolite production it is important to consider the capacity of an entire population rather than individual isolates. Knowing that populations of *P. aeruginosa* are phenotypically and genetically heterogeneous in the CF lung (Smith et al., 2006; Mowat et al., 2011; Workentine et al., 2013) and that adaptive mutations may impact the production of toxins, testing a large number of isolates is likely the only valid *in vitro* approach to demonstrate an overall lack of killing at the population level. Regional diversification of *P. aeruginosa* occurs over time in the CF lung (Jorth et al., 2015) which could potentially lead to areas where active mixed infections with *Burkholderia* would be possible while in other areas of the lung this may not happen due to HCN-independent and -dependent competition. However, due to the nature of sputum collection, it is for now impossible to determine whether the isolates come from one or several regions of the lungs. Using 26 *P. aeruginosa* clinical isolates collected over a period of 10 months from a single patient, we demonstrated that long-term adaptation of *Pseudomonas* to the CF lung had profound effects on the viability of *Burkholderia* in optimal killing LB co-cultures (Figure 3). In fact, *B. cenocepacia* became the dominant organism within the community with half of the tested CF host-adapted *P. aeruginosa* isolates, demonstrating that competitive genetic factors may be under selection in host environments. These results may explain, in part, why Bcc species dominate the *P. aeruginosa* population in certain co-infected CF patients (Schwab et al., 2014; Carmody et al., 2015), which may coincide with a reduced overall *Pseudomonas* toxicity at the population level. Loss-of-function mutations in QS genes are common in host-adapted *P. aeruginosa* (Smith et al., 2006; Hoffman et al., 2009; Cullen and McClean, 2015) and the reduced toxicity of Δ*lasR, rhlR*, and Δ*pqsA* in co-cultures (Figure 2A and Figure S7) precisely re-confirm their role in interspecies competition (Park et al., 2005; Hoffman et al., 2006; Filkins et al., 2015; Smalley et al., 2015). In addition, host adapted isolates may have additional competitive mechanism(s) not identified in this study since one isolate was fully toxic in co-culture but produced relatively low levels of HCN (Figure 3).

There is a possible association between siderophores and *Burkholderia* killing since pyoverdine was shown to have antibiotic activity against *B. cenocepacia* (Costello et al., 2014). However, Tyrrell et al. (2015) elegantly demonstrated that growth inhibition of *B. cenocepacia* by pyoverdine was likely related to its iron chelating ability rather than direct killing as previously proposed (Costello et al., 2014), which would be in agreement with our co-culture data with the mutants *pvdS* and *pvdL* (Figure S11). Pyoverdine is regulated by the PQS system (Costello et al., 2014), but the lack of *B. cenocepacia* survival with *pvdS* and *pvdL* mutants cannot explain that Δ*pqsA* and Δ*pqsR* were less toxic in co-cultures although still positive for HCN (Figure 2). Other than phenazines (Figure 2 and Figure S1), the reduced killing phenotype of Δ*pqsA* and Δ*pqsR* remains unknown since purified PQS and HHQ have no antibiotic activity against *B. cenocepacia* (Costello et al., 2014). Although pyoverdine does not seem to act as an antibiotic on *B. cenocepacia*, it would be interesting to determine the role of iron-mediated competition in the establishment of long-term complex communities *in vitro*. Investigating siderophore-mediated interactions would be highly relevant since siderophores are important virulence factors of both *P. aeruginosa* and Bcc during infections. Further, pyoverdine induces expression of genes coding for the Bcc siderophore ornibactin (Tyrrell et al., 2015), while ornibactin induces gene expression in *P. aeruginosa* (Weaver and Kolter, 2004) demonstrating that iron competition has the potential to shape communities and impact pathogenesis.

In polymicrobial communities, other microorganisms may have a direct impact on competition between two species and consequently neutralize what would typically be observed in simple *in vitro* two-species systems. Since *P. aeruginosa* uses multiple toxins to kill *B. cenocepacia*, we had to reduce the number of negative interactions to one in order to adequately evaluate the effect of a third partner. Consequently, we replaced *P. aeruginosa* by cyanide (KCN) and evaluated the impact of non-toxic cyanide-resistant bacteria on the viability of *B. cenocepacia*. The addition of non-toxic cyanide-resistant bacterial species like *P. aeruginosa rhlR* or *S. aureus* increased the tolerance of *B. cenocepacia* K56-2 to cyanide in LB co-cultures (Figures 4A,B) while less tolerant bacteria like *S. maltophilia* had a lesser impact on cyanide tolerance (Figure 4C). Interestingly, the increased cyanide tolerance conferred by *P. aeruginosa* or *S. aureus* was the result of extracellular heat labile molecule(s) that act antagonistically with cyanide (Figure 5) and consequently allowing *B. cenocepacia* to grow in the presence of cyanide concentrations typically toxic in monoculture settings (Figures 4, 5). Studies are currently underway to identify the sought after molecule(s) conferring cyanide tolerance from both *S. aureus* and *P. aeruginosa*. Neutralization of antibiotics by a third species can attenuate the inhibitory interactions between two species allowing ecological stability in well-mixed environments (Kelsic et al., 2015) while typical rock-paper-scissors interactions support microbial diversity in spatial and local environments, but not in mixed environments (Kerr et al., 2002). Our results with cyanide-resistant bacteria inhibiting negative interactions between *B. cenocepacia* and cyanide support a model where neutralization of a toxin in communities can maintain microbial diversity in well-mixed environments.

In conclusion, polymicrobial communities can involve a large number of positive and negative interactions. Here, we show that negative interactions mediated by the HCN operon of *P. aeruginosa* or cyanide in co-cultures with *B. cenocepacia* are rather complex. In fact, we show that the susceptibility of *Burkholderia* to cyanide is not only culture medium-dependent, but also reduced or even blocked via heat labile molecule(s) when non-toxic cyanide-resistant bacteria or sub-inhibitory concentrations of their metabolites are present within the community. Furthermore, within a population of *P. aeruginosa* from a single patient sample we observed a spectrum of interactions by doing pairwise co-cultures where cyanide production was not associated with killing, reinforcing the fact that cyanide is necessary, but not sufficient for killing and that extracellular factors mainly RhlR-dependent (Figure 2A) are required with cyanide for effective *Burkholderia* killing *in vitro*. Altogether, these demonstrate the complexity of interspecies competition and the unlimited numbers of possible interactions that may directly influence microbial diversity in the community. This work paves the way for future studies to dissect the role of *P. aeruginosa*-mediated competition in the establishment and development of chronic mixed infections with *Burkholderia*. Understanding these competitive interactions at the molecular level and their impact *in vivo* as well as how *Burkholderia* is more tolerant in chemically defined media will be essential for the implementation of new strategies for the treatment and management of lower airway polymicrobial infections.

## Author contributions

SB designed the study, performed experiments, and wrote the manuscript. MW performed the HCN experiment on the *P. aeruginosa* clinical isolates. XL extracted and purified the rhamnolipids. NM and GO helped in the design of the study. MS designed the study and wrote the manuscript.

### Conflict of interest statement

The authors declare that the research was conducted in the absence of any commercial or financial relationships that could be construed as a potential conflict of interest.
