# Supplementary material for: Cyanide Toxicity to Burkholderia cenocepacia Is Modulated by Polymicrobial Communities and Environmental Factors
Source: Front Microbiol. 2016 May 18;7:725. doi: 10.3389/fmicb.2016.00725 (PMC4870242; doi:10.3389/fmicb.2016.00725)
Supplement: Supplementary file 1 [file Table1.PDF]

Supplementary Material

The polymicrobial context and environmental factors modulate cyanide toxicity in *Burkholderia cenocepacia*

Steve P. Bernier*, Matthew L. Workentine, Xiang Li, Nathan A. Magarvey, George A. O'Toole[3], and Michael G. Surette*

Correspondence: Corresponding Authors: sbernier19@gmail.com, surette@mcmaster.ca

Supplementary Tables

Supplementary Table 1. Microbial strains, plasmids, and oligonucleotide primers used in this study.

Strain or Plasmid	Relevant characteristics – genotype	Reference or Source
Strains		
E. coli		
DH5α	F$^-$ *endA1 glnV44 thi-1 recA1 relA1 gyrA96 deoR nupG* Φ80d*lacZ*ΔM15 Δ(*lacZYA-argF*)U169, *hsdR17*(r_K^- m_K^+), λ–	Invitrogen
TOP10	F- *mcrA* Δ(*mrr-hsdRMS-mcrBC*) φ80*lacZ*ΔM15 Δ*lacX74 nupG recA1 araD139* Δ(ara-leu)7697 *galE15 galK16 rpsL*(Sm^R) *endA1* λ$^-$	Invitrogen
HB101	F$^-$ *mcrB mrr hsdS20*(r_B^- m_B^-) *recA13 leuB6 ara-14 proA2 lacY1 galK2 xyl-5 mtl-1 rpsL20*(Sm^R) *glnV44* λ$^-$	
SM10 λpir	Km^R *thi-1 thr leu tonA lacY supE recA*::RP4-2-Tet::Mu	Lab collection
S. cerevisiae INV*Sc*1	*MATa his3Δ1 leu2 trp1-289 ura3-52*	Invitrogen
S. aureus RN6390	Laboratory strain	(Cassat et al., 2006)
S. maltophilia K279a	Clinical isolate	(Avison et al., 2000)
P. aeruginosa		
PA14	Wild-type (WT); Burn patient isolate	(Rahme et al., 1995)
Δ*lasR*	PA14Δ*lasR*; In-frame deletion of *lasR*	(Hogan et al., 2004)
rhlR	PA14*rhlR*::Tet; Gene replacement of *rhlR*; Tet^R	(Hogan and Kolter, 2002;Hogan et al., 2004)
Δ*pqsA*	PA14Δ*pqsA*; In-frame deletion of *pqsA*	D. A. Hogan and (Ha et al., 2011)
Δ*pqsH*	PA14Δ*pqsH*; In-frame deletion of *pqsH*	(Cugini et al., 2010)
Δ*pqsR*	PA14Δ*pqsR*; In-frame deletion of *pqsR*	(Cugini et al., 2010)
Δ*phz*	PA14Δ*phzA1-G1*Δ*phzA2-G2*; In-frame deletion of the two *phzA-G* operons	(Gibson et al., 2009)
Δ*rhlA*	PA14Δ*rhlA*::Gm; Internal deletion of *rhlA*; Gm^R	(Rahim et al., 2001;Pukatzki et al., 2002;Caiazza et al., 2005)
Δ*hcnABC*	PA14Δ*hcnABC*	This study
Δ*lasR*Δ*hcnABC*	PA14Δ*lasR*Δ*hcnABC*	This study
*rhlR*Δ*hcnABC*	PA14*rhlR*::TetΔ*hcnABC*	This study
Δ*pqsA*Δ*hcnABC*	PA14Δ*pqsA*Δ*hcnABC*	This study
pvdL	PA14*pvdL*::Tn*M*; mutant ID 24971	(Liberati et al., 2006)
pvdS	PA14*pvdS*::Tn*M*; mutant ID 34241	(Liberati et al., 2006)
PAO1	Laboratory strain	Lab collection
Δ*hcnABC*	PAO1Δ*hcnABC*	This study

Clinical isolates	*P. aeruginosa* PES isolates from airways of a CF single patient	(Workentine et al., 2013)
B. cepacia		
ATCC 25416	Onion isolate (USA)	(Mahenthiralingam et al., 2000)
ATCC 17759	Soil isolate (Trinidad)	(Mahenthiralingam et al., 2000)
LMG 17997	Urinary tract infection isolate (UTI; Sweden)	(Mahenthiralingam et al., 2000)
CEP509	CF sputum isolate (Australia)	(Mahenthiralingam et al., 2000)
B. multivorans		
C1576	CF sputum isolate (UK)	(Mahenthiralingam et al., 2000)
C5393	CF sputum isolate (Canada)	(Mahenthiralingam et al., 2000)
CF-A1-1	CF sputum isolate (UK)	(Mahenthiralingam et al., 2000)
LMG 13010	CF sputum isolate (Belgium)	(Mahenthiralingam et al., 2000)
ATCC 17616	Soil isolate (USA)	(Mahenthiralingam et al., 2000)
249-2	Laboratory isolate (USA)	(Mahenthiralingam et al., 2000)
JTC	Chronic granulomatous disease (CGD) isolate (USA)	(Mahenthiralingam et al., 2000)
C1962	Clinical isolate (UK)	(Mahenthiralingam et al., 2000)
B. cenocepacia		
WT K56-2	Wild-type; CF sputum isolate (Canada)	(Mahenthiralingam et al., 2000;Varga et al., 2013)
J2315	CF sputum isolate (UK)	(Mahenthiralingam et al., 2000;Holden et al., 2009)
BC7	CF sputum isolate (Canada)	(Mahenthiralingam et al., 2000;Varga et al., 2013)
C5424	CF sputum isolate (Canada)	(Mahenthiralingam et al., 2000)
PC184	CF sputum isolate (USA)	(Mahenthiralingam et al., 2000)
H111	CF sputum isolate (Germany)	(Huber et al., 2001;Carlier et al., 2014)
HI2424	Soil isolate (USA)	(LiPuma et al., 2002)
ATCC 17765	UTI isolate (UK)	(Mahenthiralingam et al., 2000)
CEP511	CF sputum isolate (Australia)	(Mahenthiralingam et al., 2000)
J415	CF sputum isolate (UK)	(Mahenthiralingam et al., 2000)
C1394	CF sputum isolate (Canada)	(Mahenthiralingam et al., 2000)
C6433	CF sputum isolate (Canada)	(Mahenthiralingam et al., 2000)
B. stabilis		
LMG 14294	CF sputum isolate (Belgium)	(Mahenthiralingam et al., 2000)
LMG 14086	Respiratory isolate (UK)	(Mahenthiralingam et al., 2000)
B. vietnamiensis		
PC259	CF sputum isolate (USA)	(Mahenthiralingam et al., 2000)
FC441	CGD isolate (Canada)	(Mahenthiralingam et al., 2000)
LMG 16232	CF sputum isolate (Sweden)	(Mahenthiralingam et al., 2000)
B. dolosa		
LO6	CF sputum isolate	D. P. Speert
LMG 18943	CF sputum isolate (USA)	(Coenye et al., 2003)
PC543	CF sputum isolate (USA)	(Coenye et al., 2001;Workentine et al.,

		2014)
B. ambifaria		
CEP0996	CF sputum isolate (Australia)	(Coenye et al., 2003)
ATCC 53266	Soil isolate (USA)	(Coenye et al., 2003)
AMMD	Soil isolate (USA)	(Coenye et al., 2003)
B. anthina		
LMG 20980	Soil isolate (USA)	(Coenye et al., 2003)
LMG 20982		D. P. Speert
LMG 20983	CF sputum isolate (UK)	(Coenye et al., 2003)
LMG 16670	Rhizosphere isolate (UK)	(Coenye et al., 2003)
B. pyrrocinia		
LMG 14191	Soil isolate (Japan)	(Coenye et al., 2003)
LMG 21822	Soil isolate (USA)	(Coenye et al., 2003)
Plasmids		
pMQ30	Allelic replacement vector; Gm^R *sacB URA3 CEN6/ARSH4 lacZα*	(Shanks et al., 2006)
pMQ72	P_{Bad} expression vector; Gm^R	(Shanks et al., 2006)
pMQ30*hcnABC*KO	*hcnABC* knockout construct in pMQ30	This study
pMQ72*hcnABC*	P_{Bad} construct expressing *hcnABC*; Gm^R	This study
Primers		
M13F	GTAAAACGACGGCCAGT	Lab collection
M13R	CAGGAAACAGCTATGAC	Lab collection
hcn1-5L	CCAAGCTTGCATGCCTGCAGGTCGACTCTAGAGGATCCCCACGGTATTCGTCTGCCTGGC	This study
hcn1-3	GATTTACCCCTCGCGGACCTTGCCCTTTCATCCGTGAGAG	This study
hcn2-5	CTCTCACGGATGAAAGGGCAAGGTCCGCGAGGGGTAAATC	This study
hcn2-3L	AACAGCTATGACCATGATTACGAATTCGAGCTCGGTACCCGCTTTCCGAAAGGTCCGCGA	This study
hcnV5	CTTCGATCAGCGTAGTGACG	This study
hcnV3	TCGGAGCCATGAGTATAGCG	This study
hcnABC-3L	TACCCGTTTTTTTGGGCTAGCGAATTCGAGCTCGGTACCCTCTCTCACGGATGAAAGGGC	This study
hcnABC-5L	CCAAGCTTGCATGCCTGCAGGTCGACTCTAGAGGATCCCCTCGGAGCCATGAGTATAGCG	This study
