# Supplementary material for: Cyanide Toxicity to Burkholderia cenocepacia Is Modulated by Polymicrobial Communities and Environmental Factors
Source: Front Microbiol. 2016 May 18;7:725. doi: 10.3389/fmicb.2016.00725 (PMC4870242; doi:10.3389/fmicb.2016.00725)
Supplement: Supplementary file 2 [file Figure1.PDF]

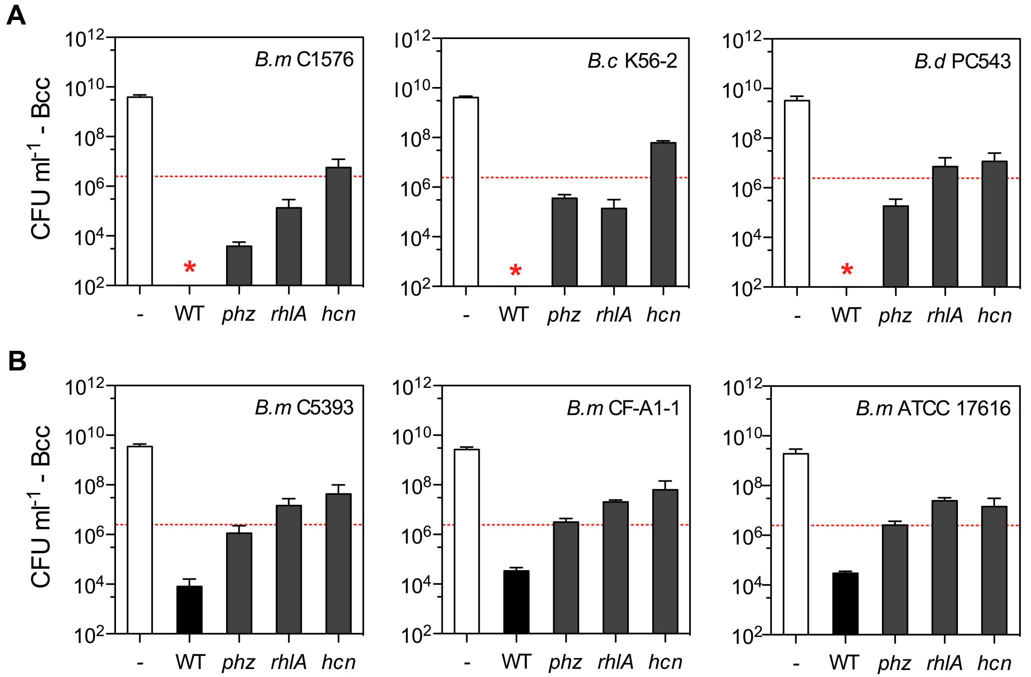

Supplementary Figure 1. QS-regulated small molecules are associated with *P. aeruginosa* toxicity. QS-regulated small molecules deficient *P. aeruginosa* PA14 mutant strains Δ*phz* (*phz*: phenazine negative)*,* Δ*rhlA* (*rhlA*: rhamnolipids negative)*,* Δ*hcnABC* (*hcn*; HCN negative) compared to their wild-type (WT) parent strain *P. aeruginosa* PA14 when co-cultured in LB medium with **(A)** highly susceptible Bcc strain to *P. aeruginosa* PA14 toxicity in co-cultures (**Figure S3A**) and **(B)** semi-tolerant Bcc strains (**Figure S3A**). Viability of *Burkholderia* was monitored via CFUs after 24 hrs in monoculture (white bars) and in mixed cultures (grey bars). Data reported represent the mean ± SD of a minimum of three replicates. Red star represents the absence of *Burkholderia* CFU recovered from the co-cultures or below the detection limits. Dotted red line represents *Burkholderia* CFUs at time 0 (~ 2 x 10^6 CFU ml^{-1}). *B.m*; *B. multivorans*, *B.c; B. cenocepacia*, and *B.d*; *B. dolosa*.
