# Supplementary material for: Cyanide Toxicity to Burkholderia cenocepacia Is Modulated by Polymicrobial Communities and Environmental Factors
Source: Front Microbiol. 2016 May 18;7:725. doi: 10.3389/fmicb.2016.00725 (PMC4870242; doi:10.3389/fmicb.2016.00725)
Supplement: Supplementary file 4 [file Figure3.PDF]

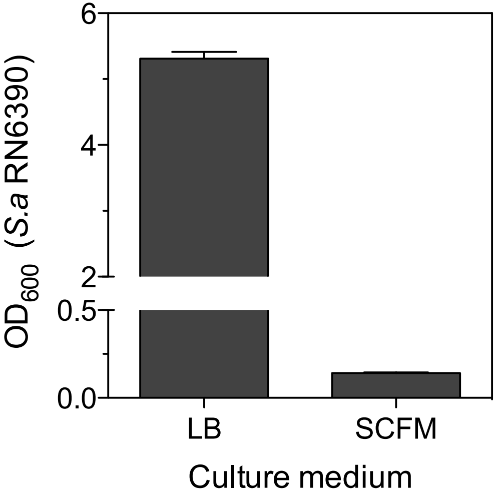

Supplementary Figure 3. Growth of *S. aureus* in SCFM compared to LB. *S. aureus* RN6390 was grown at 37°C with shaking (175 rpm) for 20 h in LB and SCFM media (5 ml; test tubes). OD_{600} was used as measurement of growth.
