# Supplementary material for: Cyanide Toxicity to Burkholderia cenocepacia Is Modulated by Polymicrobial Communities and Environmental Factors
Source: Front Microbiol. 2016 May 18;7:725. doi: 10.3389/fmicb.2016.00725 (PMC4870242; doi:10.3389/fmicb.2016.00725)
Supplement: Supplementary file 5 [file Figure4.PDF]

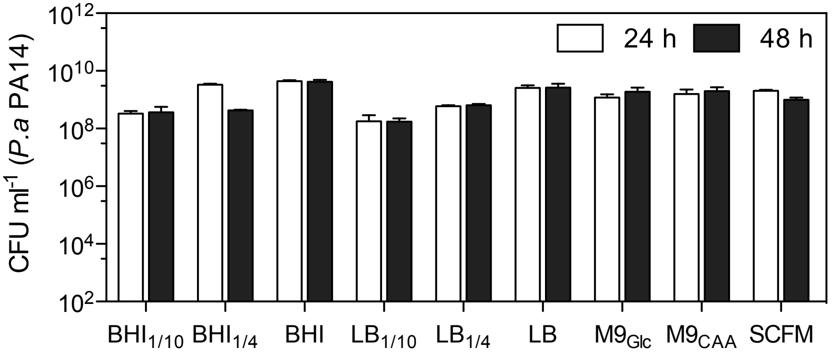

Supplementary Figure 4. Growth of *P.* aeruginosa in mixed cultures with *B. cenocepacia*. Viable populations of *P. aeruginosa* PA14 in co-culture in shaken flasks with *B. cenocepacia* k56-2 in 9 different culture media over a period of 48 h. The viability of *P. aeruginosa* PA14 was monitored via CFUs after 24 and 48 h. Data reported represent the mean ± SD of three replicates.
