# Supplementary material for: Cyanide Toxicity to Burkholderia cenocepacia Is Modulated by Polymicrobial Communities and Environmental Factors
Source: Front Microbiol. 2016 May 18;7:725. doi: 10.3389/fmicb.2016.00725 (PMC4870242; doi:10.3389/fmicb.2016.00725)
Supplement: Supplementary file 6 [file Figure5.PDF]

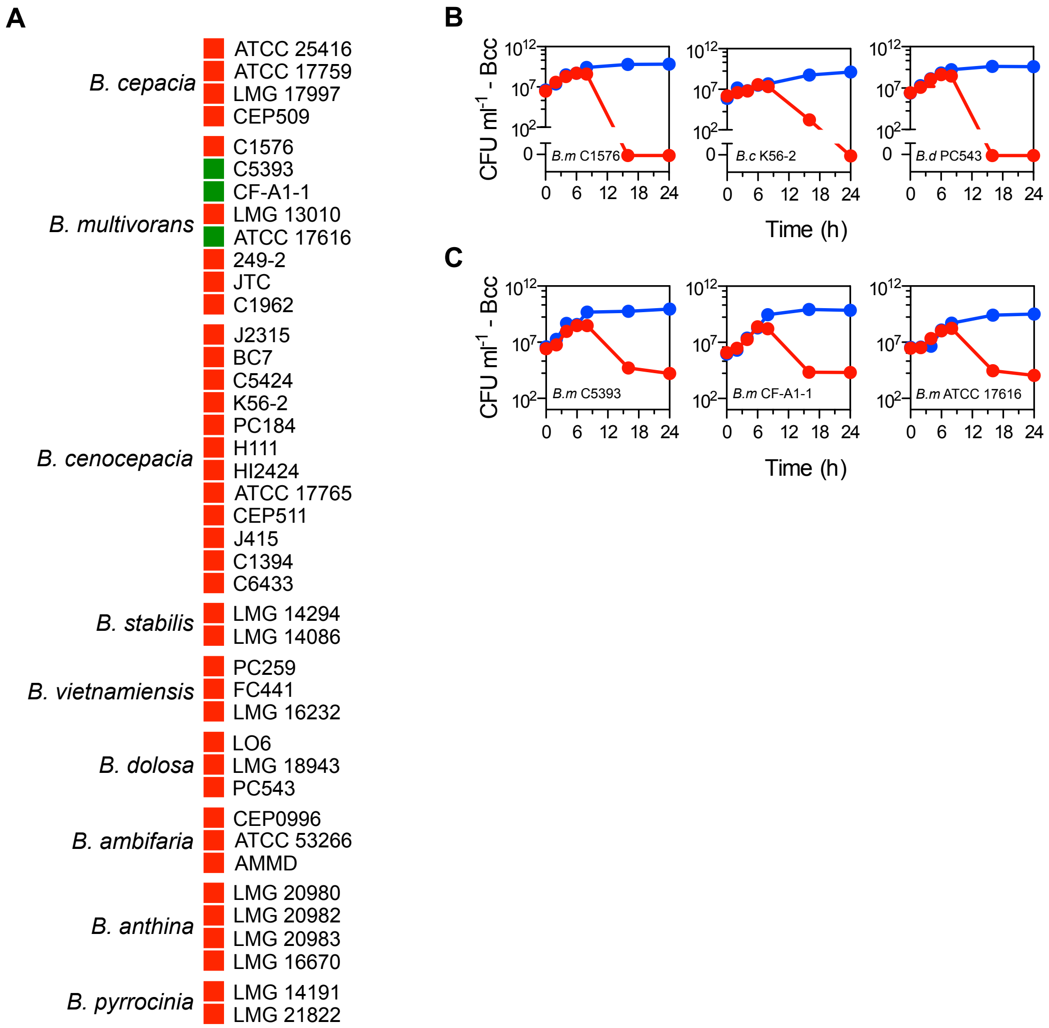

Supplementary Figure 5. The antagonistic activity of *P. aeruginosa* against Bcc species in mixed planktonic communities. (A) Survival of 41 strains representing nine Bcc species after 24 h in mixed cultures in LB medium with *P. aeruginosa* PA14 where Bcc viability is represented by green (alive) or red (dead) squares. Growth dynamics of representative sensitive **(B)** and semi-tolerant **(C)** Bcc strains from panel **A** in monoculture (blue line) or in co-culture with *P. aeruginosa* (strain PA14; red line) in shaken flasks for 24 h in LB medium. Bcc viability was monitored by CFU counts on selective agar-containing media. Data reported represent the mean ± SD of three replicates. *B.m*, *B. multivorans*; *B.c*, *B. cenocepacia*; *B.d*, *B. dolosa*.
