# Supplementary material for: Cyanide Toxicity to Burkholderia cenocepacia Is Modulated by Polymicrobial Communities and Environmental Factors
Source: Front Microbiol. 2016 May 18;7:725. doi: 10.3389/fmicb.2016.00725 (PMC4870242; doi:10.3389/fmicb.2016.00725)
Supplement: Supplementary file 7 [file Figure6.PDF]

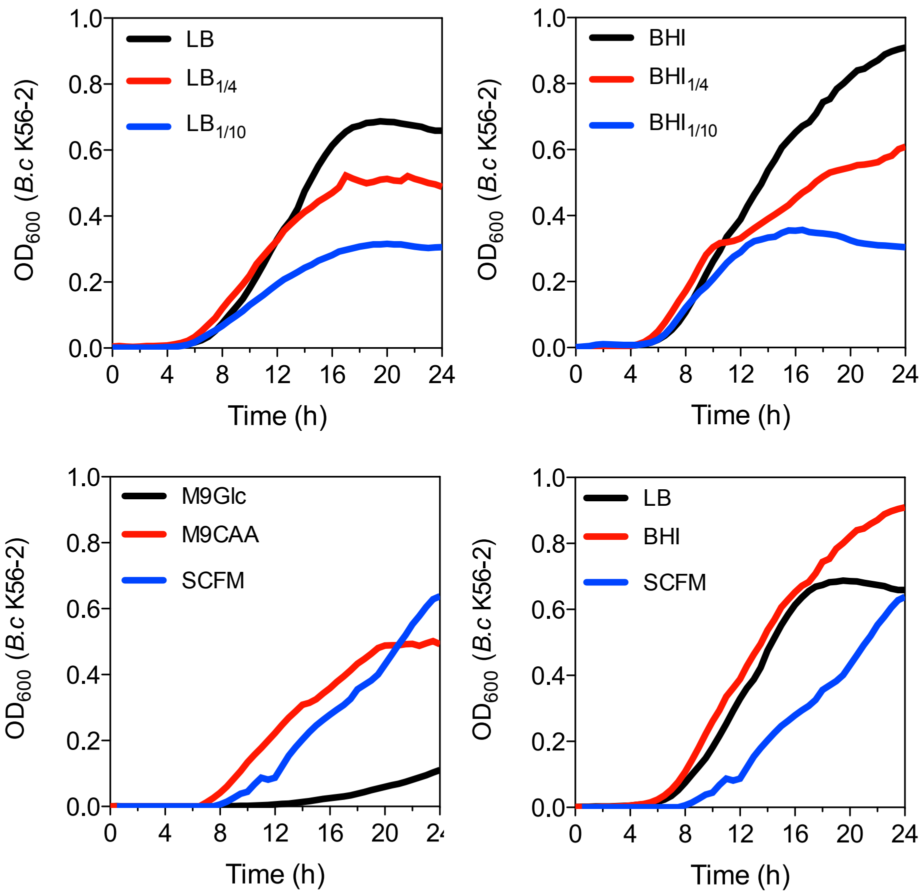

Supplementary Figure 6. Growth of *B. cenocepacia* in different culture media. *B. cenocepacia* K56-2 was grown in different culture media at 37°C with constant shaking for 24 h using 96-well plate format. Mineral oil was added to prevent evaporation and measurements were taken every 30 minutes. Data reported represent the mean of three replicates.
