# Supplementary material for: Cyanide Toxicity to Burkholderia cenocepacia Is Modulated by Polymicrobial Communities and Environmental Factors
Source: Front Microbiol. 2016 May 18;7:725. doi: 10.3389/fmicb.2016.00725 (PMC4870242; doi:10.3389/fmicb.2016.00725)
Supplement: Supplementary file 8 [file Figure7.PDF]

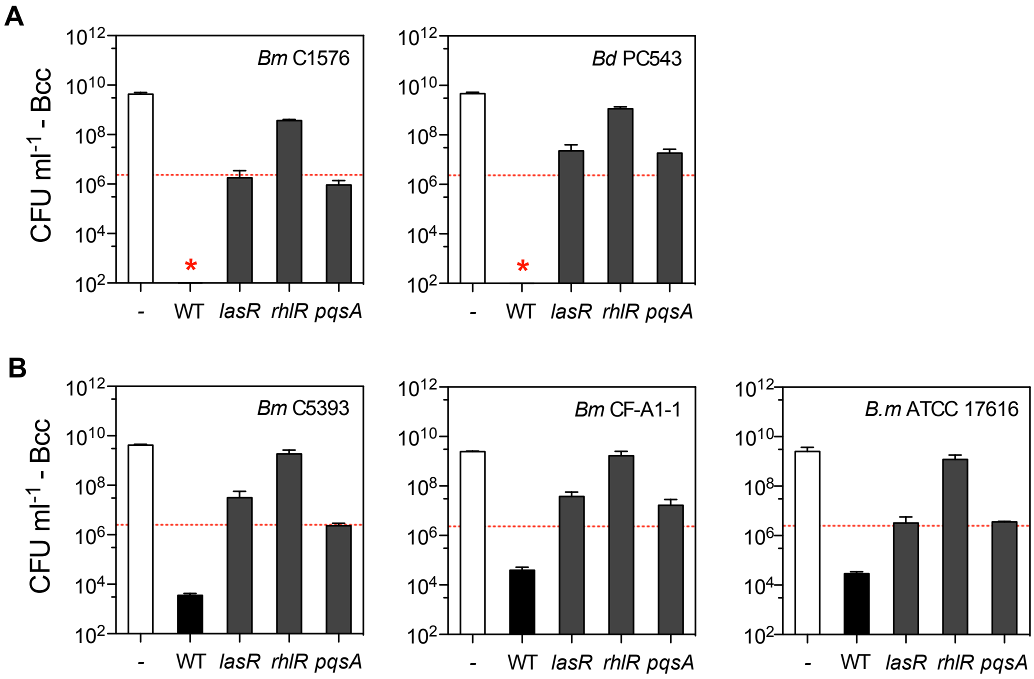

Supplementary Figure 7. Disruption of cell-cell signalling networks allows Bcc to survive in mixed communities. Sensitive **(A)** and semi-tolerant **(B)** Bcc strains were grown in monoculture (white bars) and in co-culture with wild-type (WT) *P. aeruginosa* PA14 (black bars) and its isogenic QS mutants *lasR*, *rhlR*, and *pqsA* (grey bars) in LB medium for 24 h in shaken flasks. Bcc viability was monitored by CFU counts on selective agar-containing media. Red stars in panel **A** represent the absence of Bcc CFUs recovered from the co-cultures or below the detection limit. Dotted red lines represent Bcc CFUs at time 0 (~ 2 x 10^6 CFU ml^{-1}). Data reported represent the mean ± SD of at least three replicates. *Bd, B. dolosa; Bm*, *B. multivorans*.
