# Supplementary material for: Cyanide Toxicity to Burkholderia cenocepacia Is Modulated by Polymicrobial Communities and Environmental Factors
Source: Front Microbiol. 2016 May 18;7:725. doi: 10.3389/fmicb.2016.00725 (PMC4870242; doi:10.3389/fmicb.2016.00725)
Supplement: Supplementary file 9 [file Figure8.PDF]

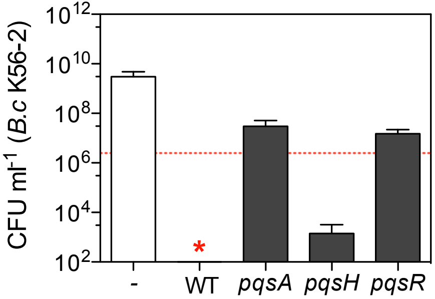

Supplementary Figure 8. Role of the PQS system in mixed cultures. Viability of *B. cenocepacia* K56-2 after 24 h in mixed cultures WT *P. aeruginosa* PA14 and derivative mutants in genes involved with the PQS signalling system with Δ*pqsA (*PQS negative, HHQ negative), Δ*pqsH (*PQS negative, HHQ positive), and Δ*pqsR (*PQS negative, HHQ negative). *Burkholderia* viability was monitored by CFU counts on selective agar-containing media. Dotted red line represents *Burkholderia* CFUs at time 0 (~ 2 x 10^6 CFU ml^{-1}) and the red star represents the absence of *Burkholderia* CFU recovered from the co-cultures or below the detection limits. Data reported represent the mean ± SD of three replicates.
