# Supplementary material for: Cyanide Toxicity to Burkholderia cenocepacia Is Modulated by Polymicrobial Communities and Environmental Factors
Source: Front Microbiol. 2016 May 18;7:725. doi: 10.3389/fmicb.2016.00725 (PMC4870242; doi:10.3389/fmicb.2016.00725)
Supplement: Supplementary file 10 [file Figure9.PDF]

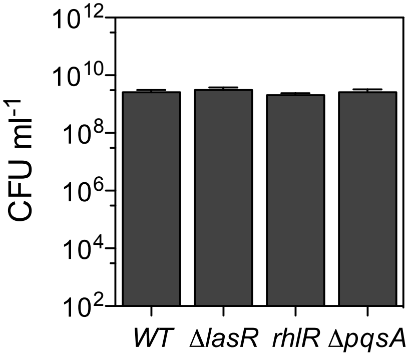

Supplementary Figure 9. Viability of *P. aeruginosa* QS mutants in co-cultures with *B. cenocepacia*. Viable populations of *P. aeruginosa* PA14 (WT) and its QS mutant derivatives in co-culture in shaken flasks with *B. cenocepacia* k56-2 in LB medium over 24 h. The viability was monitored via CFUs. Data reported represent the mean ± SD of three replicates.
