# Supplementary material for: Cyanide Toxicity to Burkholderia cenocepacia Is Modulated by Polymicrobial Communities and Environmental Factors
Source: Front Microbiol. 2016 May 18;7:725. doi: 10.3389/fmicb.2016.00725 (PMC4870242; doi:10.3389/fmicb.2016.00725)
Supplement: Supplementary file 11 [file Figure10.PDF]

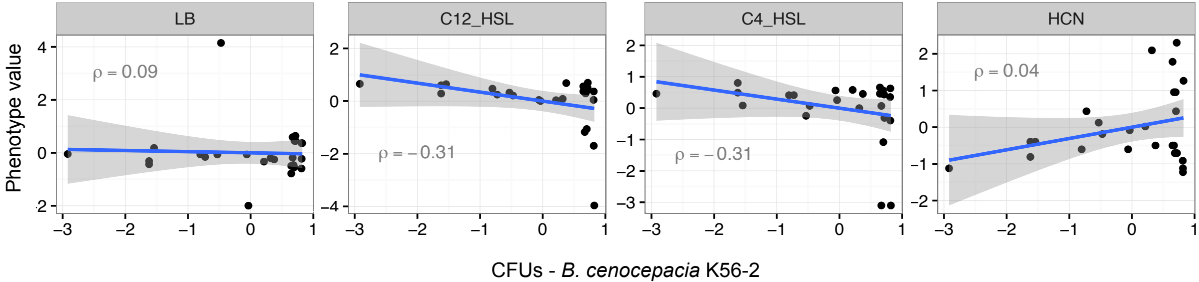

Supplementary Figure 10. Spearman rank correlation for *B. cenocepacia* CFUs. Phenotypic data for the *P. aeruginosa* clinical isolates for QS signals (C4- and C12-HSL) and growth in LB were obtained from (Workentine et., 2013). HCN and *B. cenocepacia* CFUs data are those from Figure 3. Data for C4-HSL, C12-HSL, and B. cenocepacia CFUs were log-normalized prior to performing correlations. Spearman's correlation coefficient (ρ) for each phenotype is shown on each graph. No significant correlation was identified (p value > 0.05).
