# Supplementary material for: Cyanide Toxicity to Burkholderia cenocepacia Is Modulated by Polymicrobial Communities and Environmental Factors
Source: Front Microbiol. 2016 May 18;7:725. doi: 10.3389/fmicb.2016.00725 (PMC4870242; doi:10.3389/fmicb.2016.00725)
Supplement: Supplementary file 12 [file Figure11.PDF]

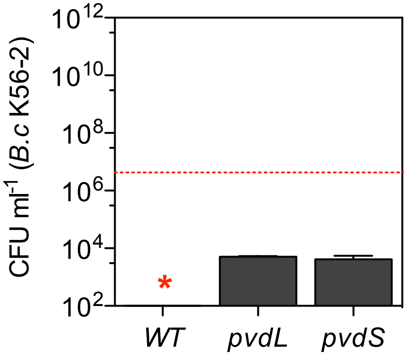


**Supplementary Figure 11. Co-cultures of *B. cenocepacia* with pyoverdine-negative *P. aeruginosa* mutants.** Viability of *B. cenocepacia* K56-2 after 24 h in mixed cultures with WT *P. aeruginosa* PA14 and its derivative mutants in genes involved in the biosynthesis of pyoverdine *pvdL* and *pvdS*. Dotted red line represents *Burkholderia* CFUs at time 0 (~ 2 x $10^6$ CFU $ml^{-1}$) and the red star represents the absence of *Burkholderia* CFU recovered from the co-cultures or below the detection limits. Data reported represent the mean ± SD of three replicates.
